# Supplementary material for: PBP4 is required for serum-induced cell wall thickening and antibiotic tolerance in Staphylococcus aureus
Source: Antimicrob Agents Chemother. 2024 Oct 21;68(11):e00961-24. doi: 10.1128/aac.00961-24 (PMC11539222; doi:10.1128/aac.00961-24)
Supplement: Supplemental material — Table S1 and Figure S1. [file aac.00961-24-s0001.pdf]

PBP4 is required for serum-induced cell wall thickening and antibiotic tolerance in *Staphylococcus aureus*

Elizabeth V. K. Ledger[1,2,3] and Ruth C. Massey[1,2,4]

[1] School of Microbiology, University College Cork, Cork, Ireland.

[2] APC Microbiome Ireland, University College Cork, Cork, Ireland.

[3] Centre for Bacterial Resistance Biology, Imperial College London, London, UK.

[4] School of Cellular and Molecular Medicine, University of Bristol, Bristol, UK.

Supplementary data file

Supplementary Table 1 and Supplementary Figure 1

Supplementary Table 1. Details of the clinical strains used in this study.

**Strain ID**	**Accession number**	***pbp4* SNP**	**HADA (RFU/OD_{600})**
ASARM61	ERR084492	No	151274.5
ASARM70	ERR084501	Synonymous 879G>A	152697.6
ASARM71	ERR084502	No	126439.4
ASARM72	ERR084503	No	150334.2
ASARM73	ERR084504	No	111233
ASARM74	ERR084505	No	159019.9
ASARM77	ERR084506	No	142518.9
ASARM76	ERR084507	No	183836.5
ASARM75	ERR084508	No	176864.6
ASARM80	ERR084509	No	211497.7
ASARM79	ERR084510	No	164836
ASARM59	ERR084493	No	165364.6
ASARM83	ERR084513	No	147809.8
ASARM84	ERR084514	No	142095.5
ASARM86	ERR084516	No	226125
ASARM87	ERR084517	No	215884.5
ASARM89	ERR084519	No	182293.2
ASARM62	ERR084494	No	177857.7
ASARM93	ERR084522	No	172872.2
ASARM95	ERR084523	No	168433.9
ASARM96	ERR084524	No	125618.6
ASARM97	ERR084525	No	169614.1
ASARM99	ERR084527	No	165651.1
ASARM100	ERR084528	No	176369.9
ASARM101	ERR084529	No	166845
ASARM102	ERR084530	No	163152.8
ASARM103	ERR084531	No	156151.4
ASARM105	ERR084533	No	161566.1
ASARM107	ERR084534	No	175022.2
ASARM109	ERR084535	No	177724.2
ASARM114	ERR084540	No	160135.4
ASARM64	ERR109523	No	110420.5
ASARM116	ERR084541	No	164537.7
ASARM117	ERR084542	No	190559.9

ASARM118	ERR084543	No	152330.6
ASARM119	ERR084544	No	180603.5
ASARM120	ERR084545	No	160555.6
ASARM121	ERR084546	No	170425.6
ASARM122	ERR084547	No	198076.5
ASARM124	ERR084548	Nonsynonymous 420 A>C	31339.64
ASARM125	ERR084549	No	163946.5
ASARM126	ERR084550	No	181816.5
ASARM65	ERR084497	No	120655.6
ASARM127	ERR084551	No	133460.6
ASARM128	ERR084552	No	164894.7
ASARM132	ERR084554	No	162184.1
ASARM133	ERR084556	No	188048.5
ASARM134	ERR084557	No	157260.9
ASARM135	ERR084558	No	182532.5
ASARM136	ERR084559	No	139611.1
ASARM137	ERR084560	No	183746.8
ASARM67	ERR084498	No	186761
ASARM138	ERR084561	No	153196.6
ASARM139	ERR084562	No	178510.9
ASARM140	ERR084563	No	176486.3
ASARM141	ERR084564	No	225584.8
ASARM142	ERR084565	No	153257.6
ASARM143	ERR084566	No	211622.6
ASARM144	ERR084567	No	149930.1
ASARM145	ERR084568	No	170581.9
ASARM68	ERR084499	No	209713.3
ASARM148	ERR084571	No	188189.5
ASARM154	ERR084575	No	155028.3
ASARM153	ERR084576	No	151903.4
ASARM155	ERR084578	No	198692.4
ASARM160	ERR084579	No	183246.7
ASARM69	ERR084500	No	152676.9
ASARM162	ERR084581	No	142612.7
ASARM164	ERR084582	No	169513.2
ASARM163	ERR084583	No	156410.4
ASARM166	ERR084584	No	162167.1
ASARM165	ERR084585	No	195943
ASARM167	ERR084586	No	183259
ASARM168	ERR084587	No	161667.5
ASARM169	ERR084638	No	179021.9

ASARM179	ERR084648	No	153559.6
ASARM181	ERR084650	No	159738.3
ASARM183	ERR084652	No	227859.1
ASARM184	ERR084653	No	197405.9
ASARM170	ERR084639	No	186022.6
ASARM191	ERR084658	No	158991.8
ASARM193	ERR084659	No	211593.2
ASARM199	ERR084664	No	169671
ASARM200	ERR084665	No	160345.9
ASARM201	ERR084666	No	168708.7
ASARM171	ERR084640	No	147242.5
ASARM203	ERR084667	No	213237.2
ASARM204	ERR084668	No	164123.9
ASARM205	ERR084669	No	178405.3
ASARM208	ERR084671	No	138694.8
ASARM209	ERR084672	No	168964.2
ASARM207	ERR084673	No	143243.2
ASARM211	ERR084675	No	137671
ASARM212	ERR084676	No	131155.5
ASARM172	ERR084641	No	169392.5
ASARMLT1	ERR084678	No	166094.2
ASARMLT2	ERR084679	No	184064.8
ASARMLT3	ERR084680	No	167359
ASARM195	ERR084714	No	155606.3
ASARM176	ERR084645	No	154856.3
ASARM177	ERR084646	No	162984.9
ASARM217	ERR171907	No	155872.8
ASARM220	ERR171908	No	176502.9
ASARM222	ERR171910	No	178102.1
ASARM223	ERR171911	No	188774.3
ASARM224	ERR171912	No	133295.3
ASARM110	ERR223125	No	172142.3
ASARM108	ERR223118	No	197180.4
ASASM42	ERR109502	No	206715.9
ASASM56	ERR109515	No	148914.5
ASASM12	ERR109476	No	163313.1
ASASM61	ERR109520	No	171618.4
ASASM64	ERR084496	No	151796.7
ASASM71	ERR109528	No	183412
ASASM73	ERR109530	No	156821.9
ASASM90	ERR109540	No	164555.9
ASASM96	ERR109546	No	150968

ASASM97	ERR109547	No	197283.9
ASASM120	ERR109567	No	83346.79
ASASM132	ERR109578	No	205175.5
ASASM138	ERR109584	No	139711.7
ASASM140	ERR109586	No	152372.3
ASASM125	ERR109572	No	131370.1
ASASM127	ERR109574	No	142147.3
ASASM181	ERR109623	No	159693
ASASM190	ERR109630	No	147543
ASASM246	ERR114858	No	186166.2
ASASM262	ERR114873	No	111483.3
ASASM390	ERR172029	No	107684.2
ASASM392	ERR172031	No	173241.6
ASASM430	ERR172068	No	154671.3
ASASM168	ERR223120	No	162396.6

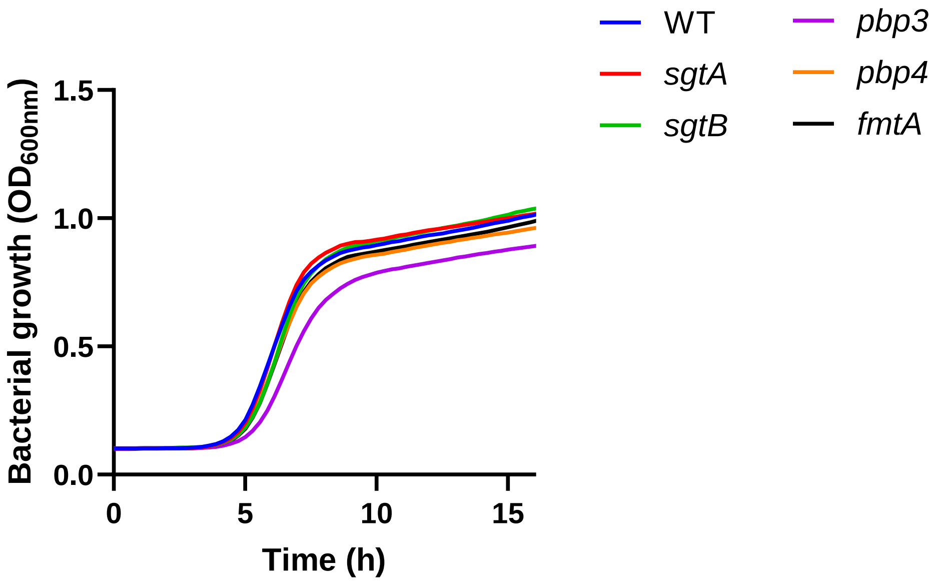

Fig. S1. The *pbp4*::Tn mutant does not have a growth defect. JE2 WT and transposon mutants in *sgtA, sgtB, pbp3, pbp4* and fmtA were diluted to 5 x 10^5 CFU ml^{-1} in fresh TSB and bacterial growth (OD_{600}) in TSB was measured every 15 min for 16 h. Data represent the mean of three independent replicates and error bars have been omitted for clarity. Data were analysed by two-way ANOVA with Dunnett's *post-hoc* test. The *pbp3* mutant was significantly lower than the WT from 6 h onwards and no other mutants were significantly different from WT at any time-point.
